# Transitioning From Room to Zoom: The 2020 Cardiovascular Fellows’ Boot Camp in the COVID-19 Era

**DOI:** 10.14797/mdcvj.1064

**Published:** 2021-12-15

**Authors:** Akanksha Thakkar, Yehia Saleh, C. Huie Lin, William Zoghbi, Nadeen Faza, Bindu Chebrolu

**Affiliations:** 1 Houston Methodist DeBakey Heart & Vascular Center, Houston Methodist Hospital, Houston, Texas, US

**Keywords:** medical education, virtual education, COVID-19, cardiology, boot camp

## Abstract

For 10 years, the annual Houston Methodist Cardiovascular Fellows’ Boot Camp hosted hundreds of cardiovascular trainees in Houston for a concise yet comprehensive 3-day training program for new fellows. The cornerstone of the program was the hands-on Skills Academy, which included a variety of timed learning stations that taught surgical techniques, dissection skills, and suturing techniques as well as echocardiography and cardiac catheterization using simulators. This was followed by 2 days of didactics covering essential topics in each specialty. However, that model was upended in 2020 by the COVID-19 pandemic. The pandemic forced the digitization of medical education and posed significant challenges as we transitioned Boot Camp to a virtual format. In this editorial, we review our experience designing and implementing a virtual cardiology track of the Houston Methodist Cardiovascular Fellows’ Boot Camp and highlight challenges and proposed solutions in the era of virtual education.

## Introduction

**Figure 1:**
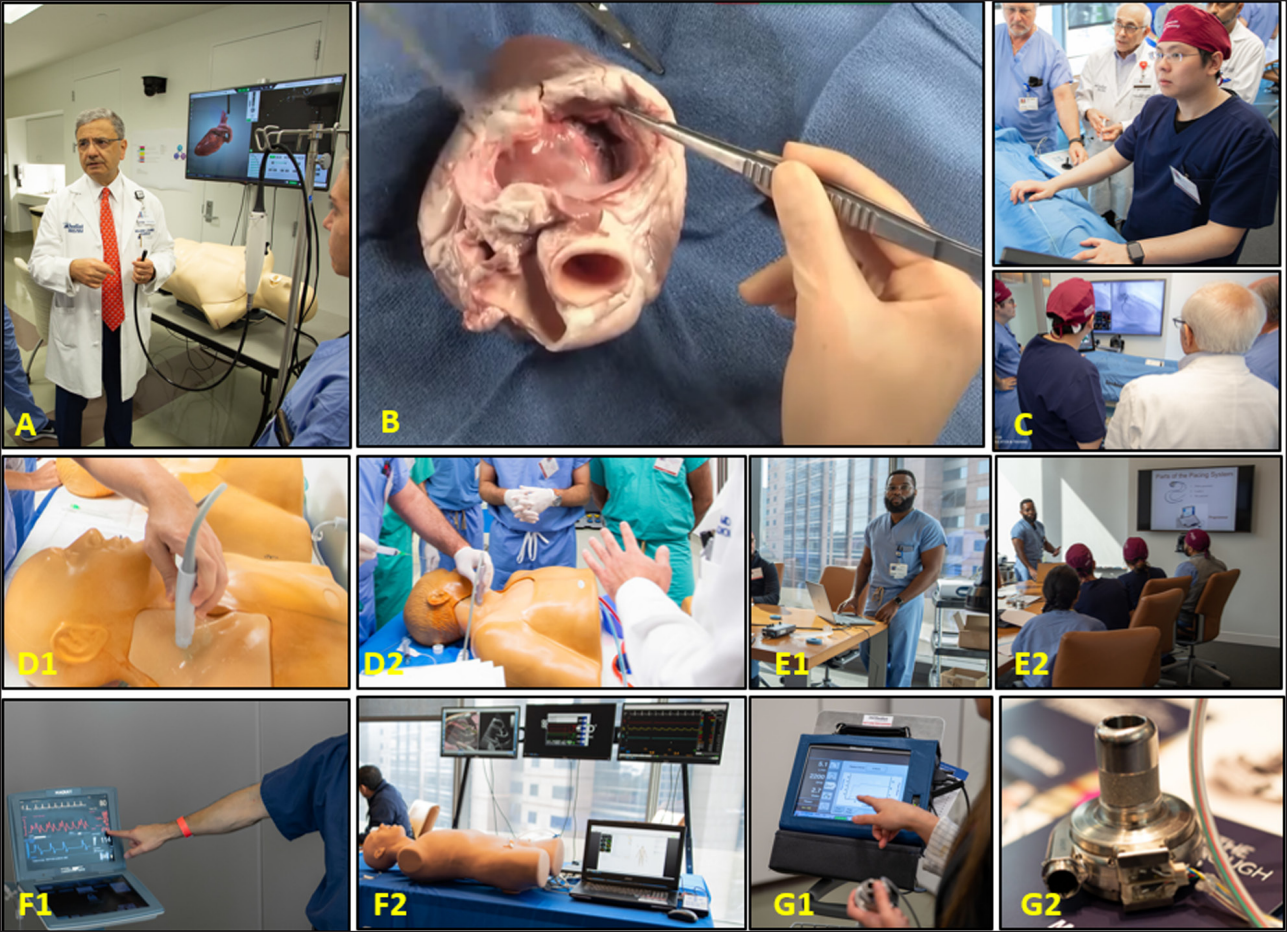
Hands-on skills stations in the prepandemic era. **(A)** Learning transesophogeal echocardiography using a simulator model. **(B)** Porcine heart dissection. **(C)** Learning cardiac catheterization skills and angiographic anatomy using a simulator model. **(D)** Practicing vascular ultrasound and ultrasound-guided access. **(E)** Learning device interrogation. (F) Understanding the basics and troubleshooting for mechanical circulatory support devices. **(G)** Learning the basic concepts of left ventricular assist device management.

**Figure 2:**
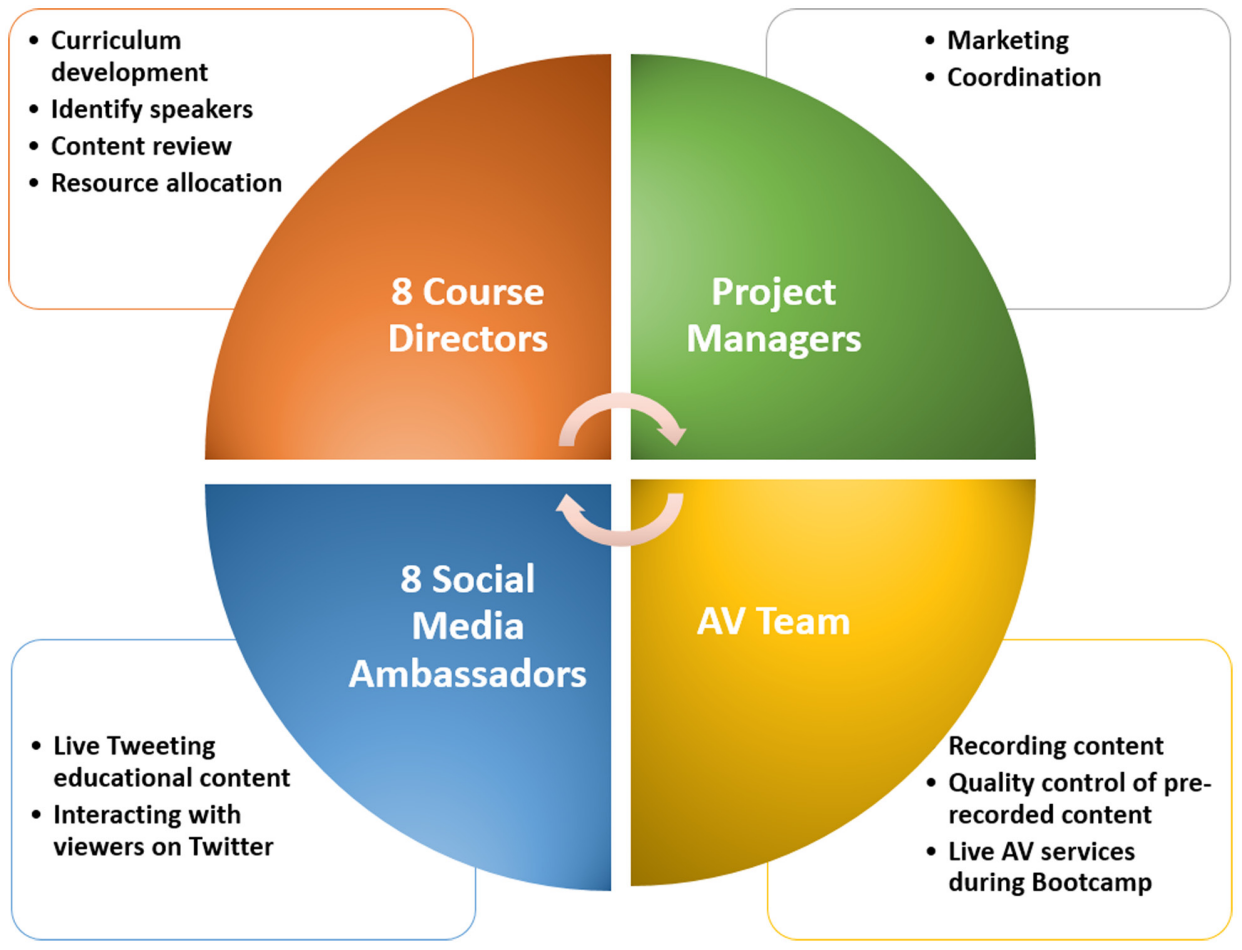
A team-based approach was used to plan and execute the virtual Boot Camp.

The Houston Methodist Cardiovascular Fellows’ Boot Camp started in 2009 as a combination of hands-on “Skills Academy” and “Didactic College” focusing on fundamentals of peripheral vascular sciences for cardiovascular surgery and interventional cardiology trainees, but over the years it has evolved into a multidisciplinary training program with tracks for cardiology, cardiac anesthesia, and cardiac and vascular surgery (***Figure 1***).^1^ In 2020, plans for the Boot Camp were upended by the coronavirus 2019 (COVID-19) pandemic, forcing our program, like so many around the world, to go virtual. In this editorial, we share our experience in organizing the first virtual edition of the Boot Camp cardiology track. Transitioning to a digital platform posed unique challenges (***Table 1***) but offered the opportunity to reach a larger audience. Similar to other programs that have transitioned to online teaching conferences and social media conversations to avoid interruptions in medical education,^2,3,4,5^ our goal was to transition the Boot Camp cardiology track from room to Zoom while preserving the program’s interactive nature. We organized a team-based approach to identify and address the unique needs of a virtual conference (***Figure 2***). Eight course directors from different subspecialties of cardiology (general cardiology, imaging, heart failure, interventional, and electrophysiology) worked on developing a curriculum, identifying and inviting speakers, reviewing content, and allocating resources.

**Table 1:** Challenges and solutions for moving Boot Camp from in-person to virtual.


Challenges	Solutions

Avoid Zoom fatigue	Concise content made available for on-demand viewing

Reduce connectivity issues	Prerecorded lectures to minimize errors in live production and onsite audiovisual team for troubleshooting

Hands-on skills lab unavailable without in-person component	Prerecorded skills sessions and live “show and tell”

Replicate in-person networking and mentorship opportunities	Hold live virtual “Meet the Expert” sessions in small-group Zoom breakout rooms

Keep the event interactive	Use Zoom chat box for real-time question-and-answer, digital moderators to monitor questions, and social media team to encourage online conversations about content


## Hands-on to Hands-off

Feedback from prior Boot Camp participants showed that the hands-on skills lab was an indispensable part of the program, so preserving the educational potential of the skills lab became our primary goal for the virtual program. Another key objective was to plan didactic sessions in a way that reduced Zoom fatigue by incorporating interaction among participants.

**Figure 3:**
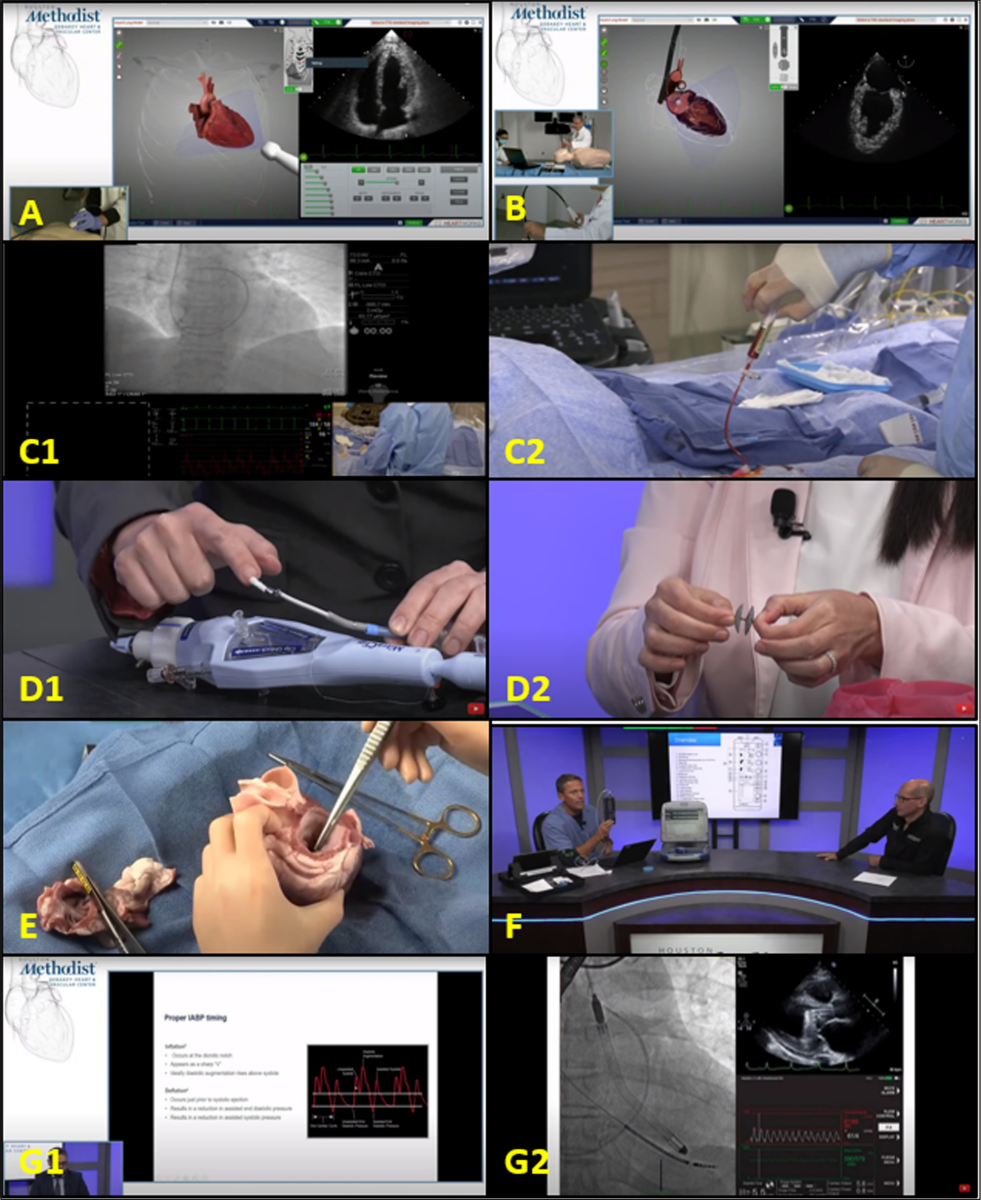
Hands-on skills session. **(A)** Transducer handling and optimal views were demonstrated simultaneously to teach how to obtain optimal transthoracic echocardiography images. **(B)** Transesophageal echocardiography was taught on a simulator model, starting with intubation technique to acquiring optimal views. **(C)** The cardiac catheterization skills demonstration included best practices to obtain vascular access and basics of performing right heart catheterization and coronary angiography. **(D)** A show-and-tell session demonstrated commonly used transcatheter devices to treat structural heart disease. **(E)** Porcine heart dissection was demonstrated on screen to explain cardiac anatomy. **(F)** Pacemaker and defibrillator basics and interrogation were demonstrated live from our studio. **(G)** A prerecorded video was used to explain the basics of mechanical circulatory support devices.

The transition from hands-on to hands-off skills sessions started with using prerecorded videos to demonstrate skills such as echocardiography, vascular access, catheterization, and porcine heart dissection. We planned a show-and-tell session to showcase some of the most frequently used transcatheter devices (***Figure 3***).

Didactic content was presented over two 6-hour days covering five subspecialty areas: coronary artery disease, cardiovascular imaging, heart failure, structural heart disease, and electrophysiology. To keep the presentations engaging, participants had the opportunity to interact with experts in real-time using the virtual conference platform’s built-in chat feature and Twitter.

## Potential to Reach a Larger Audience

Before the pandemic, fellows from across the United States had to travel to Houston to participate in Boot Camp. In 2019, we could only accommodate 54 cardiology fellows in person, so access to Boot Camp content was limited. Transitioning to a virtual platform provided the opportunity to engage a much larger audience, and 139 fellows (including international fellows) were able to participate in 2020. Streaming the content on Livestream, Twitter, Facebook, and YouTube reached an even larger audience and allowed for on-demand viewing.

## Choosing a Virtual Platform

We chose Zoom as the virtual platform because of the software’s intuitive nature and widespread use in the medical community. As noted earlier, the chat feature allowed constant interaction between learners and teachers, and the platform made it simple to switch between prerecorded content and live sessions. Also, we split participants into several groups for focused conversations during the “Meet the Experts” sessions.

## “Meeting” the Experts

The original Boot Camp included a Meet the Experts session where fellows could network with physicians in their subspecialty of interest, allowing them to exchange ideas and build relationships with potential mentors and colleagues. We organized this session using Zoom break-out rooms. Fellows were asked to enter the break-out room in their specialty of interest so they could engage in a group discussion with experts on topics such as career paths and research ideas.

## Going Live

**Figure 4:**
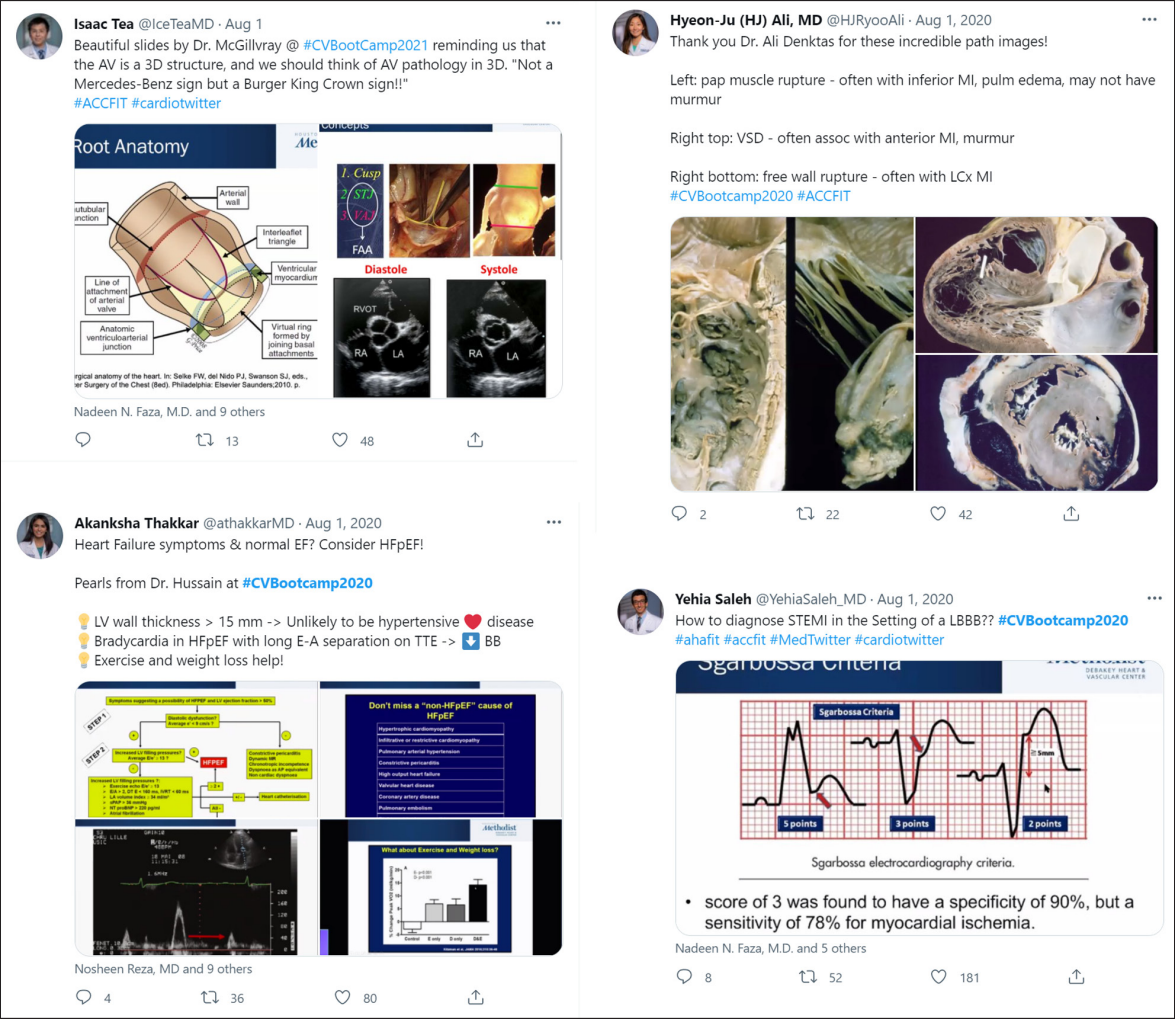
The social media team continued the conversation on Twitter by posting educational content and interacting with the audience.

Each subspecialty session had two faculty moderators (at least one on-site in the production studio) to answer questions from the audience, one digital moderator to monitor the chat box for questions, and other faculty experts logged in to the Zoom conference to interact with fellows via chat. The audiovisual team ensured smooth transitions between live interactions and prerecorded content. The social media team used Twitter to interact with fellows and post educational content from the conference using the hashtag #CVBootcamp2020 (***Figure 4***).

## Reflections

The first virtual Cardiovascular Fellows’ Boot Camp (cardiology track) stood out for its reach and educational value. Live content on other social media platforms (ie, Twitter, Facebook, and Livestream) was viewed 4,012 times on day 1 and 2,304 times on day 2. At the time of this writing, YouTube views were up to 11,000 for day 1 content and 4,700 for day 2, with 137,500 Twitter impressions on day 1.

At the end of the event, fellows filled out online program evaluations. The respondents lauded the lectures by senior fellows for their content as they tended to cover real life overnight and on-call emergencies. While the feedback was positive overall, we identified some areas for improvement. First, attendees preferred to spend more time discussing “clinical pearls” and less time talking about widely available literature. Second, attendees recommended devoting more time to areas that are less commonly addressed during internal medicine residency training, such as mechanical support devices. We analyzed the feedback in a debrief meeting and plan to incorporate these suggestions into the curriculum.

We believe that virtual conferences will remain a cornerstone in medical education even after the pandemic. In sharing our experience, we hope that other programs will continue to build on this model to educate future physicians, leaders, and scientists. Although it is impossible to replace the hands-on experience, we present an alternative learning platform that can bridge educational gaps when in-person training is not an option.
